# Imaging Features of Extra Cranial Parapharyngeal Space Meningioma: Case Report

**DOI:** 10.5812/kmp.iranjradiol.17351065.3132

**Published:** 2011-11-25

**Authors:** Kishor Taori, Nischal G. Kundaragi, Amit Disawal, Chetan Jathar, Prajwalit P. Gaur, Jawahar Rathod, Virendra Patil

**Affiliations:** 1 Department of Radiodiagnosis, Government Medical College, Nagpur Maharashtra, India; 2 Hospital Chennai, SRM University Medical College, Tamilnadu, India

**Keywords:** Meningioma, Neoplasms, Magnetic Resonance Imaging

## Abstract

Parapharyngeal tumors are less common in clinical practice and are often difficult to diagnose upon clinical examination due to the anatomic complexity of the region. We report a rare case of extracranial parapharyngeal space meningioma presenting as a cervical mass with encasement of cranial nerves giving tram track appearance and features on various imaging modalities [Radiographs, Ultrasound, Computed tomography (CT) scan and Magnetic resonance imaging (MRI)].

## 1. Introduction

Meningiomas are relatively common neoplasms of the central nervous system comprising about 18% of all primary intracranial tumors and about 25% of all primary intraspinal tumors. Extracranial meningiomas compose approximately 2% of all meningiomas, which arise from ectopic arachnoid tissue and are very rare tumors. Evaluation by computed tomography (CT) scan, Magnetic resonance imaging (MRI) and/or angiography should be considered in such conditions. Surgery is the definite management. However, radiotherapy may be performed for inoperable lesions [1]. We report a rare case of extracranial parapharyngeal space meningioma presenting as a cervical mass with encasement of major carotid vessels and cranial nerves giving the tram track appearance. Appearance on CT scan suggesting encasement of cranial nerves has not been reported in the literature. Evaluation of the cranial nerve involvement by imaging may predict the post-operative morbidity.

## 2. Case Presentation

**Figure 1:**
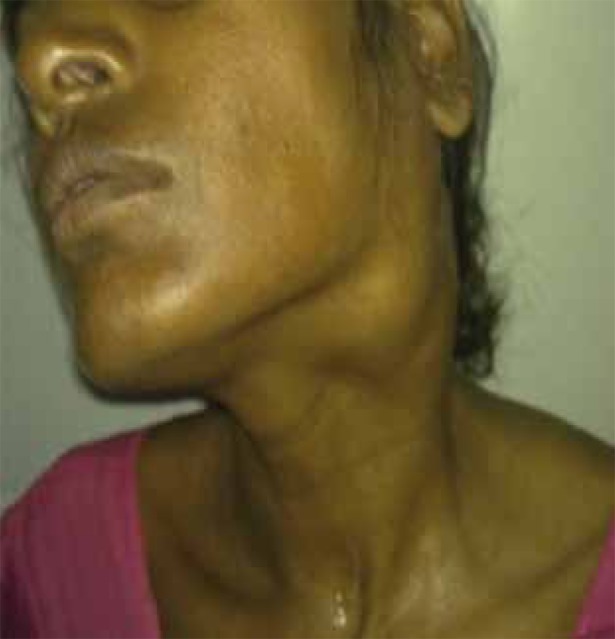
A 42-Year-Old Female, Clinical Photograph Shows Swelling on the Left Side of the Neck Posteroinferior to the Angle of Mandible.

A 42-year-old female presented with a history of painless swelling on the left side of the neck from 6 months ago with hoarseness. On clinical examination, a firm, non-tender swelling was noted in the upper part of the anterior triangle of the neck, posterior to the angle of the mandible on the left side (Figure 1) with oropharyngeal bulge and deviation of the uvula and tongue to the right side. Indirect laryngoscopy revealed restricted left vocal cord mobility indicating weakness of the tenth cranial nerve. Clinical signs suggestive of ninth and eleventh cranial nerve involvement were also noted. Family history was non-contributory.

**Figure 2:**
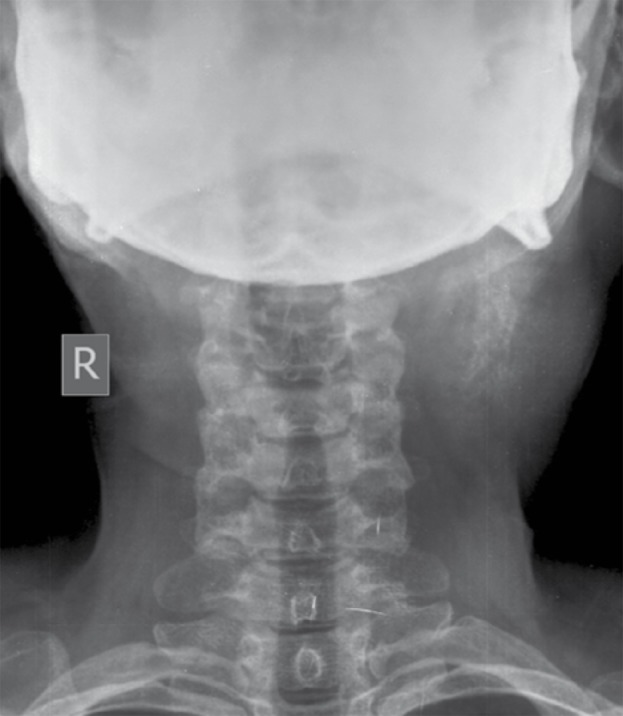
Plain Radiograph, AP View of the Neck Shows Soft Tissue Opacity on the Upper Left Side of the Neck With Central Amorphous Calcifications.

**Figure 3:**
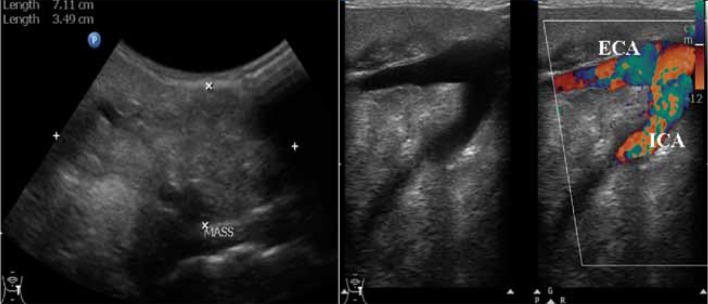
Ultrasound Shows a Well-Defined Heterogeneous Isoechoic Hypovascular Mass Lesion in Relation to the Left Parapharyngeal Region Encasing and Splaying the Carotid Vessels With Central Calcifications.

Plain radiography of the neck (Figure 2) showed well-defined inhomogeneous soft tissue opacity with central amorphous calcifications in the supero-lateral aspect of the neck on the left side. Ultrasonography was done to confirm the mass lesion revealed well defined heterogeneous isoechoic mass lesion in relation to the left parapharyngeal region encasing the carotid vessels and causing displacement of adjacent soft tissue structures with maintained fat planes. Multiple central calcific foci giving post-acoustic shadowing were noted within the mass lesion (Figure 3). Minimal vascularity was noted on color Doppler study.

**Figure 4:**
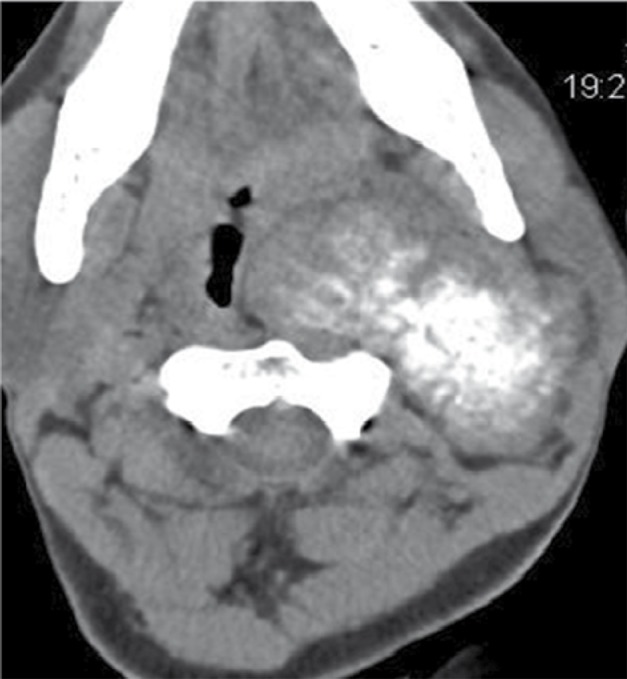
Plain CT Scan, Axial Section Revealed a Well-Defined Soft Tissue Density Mass Lesion in the Left Parapharyngeal Region With Central Dense Amorphous Calcifications.

**Figure 5:**
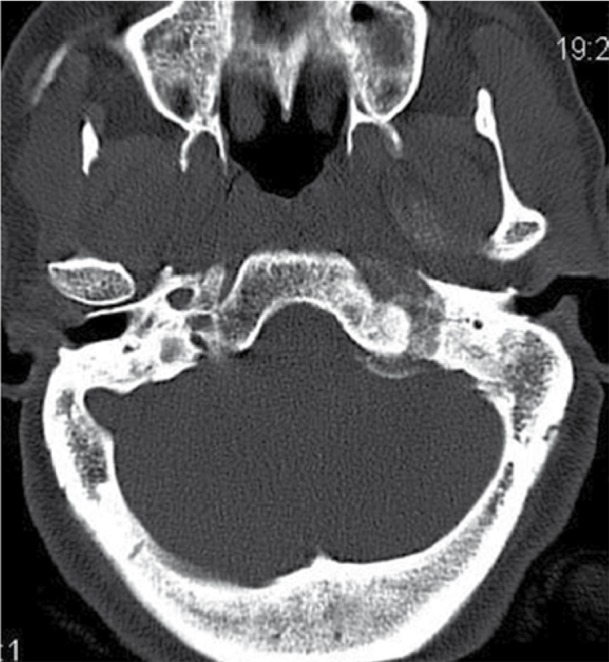
CT Scan Bone Window Shows Irregular Jugular Foramen Well- Defined Soft Tissue Density Mass Lesion in the Left Parapharyngeal Region With Central Dense Amorphous Calcifications. Small Intracranial Part is Also Noted.

Predominant left parapharyngeal isodense mass lesion showing moderate heterogeneous contrast enhancement with central amorphous and dense calcifications was noted in the CT scan of the neck (Figure 4). Superiorly, the lesion extended intracranially into the left cerebellopontine (CP) angle cistern through the left hypoglossal and jugular foramina. Encasement of the entire left internal carotid artery and a part of the left external carotid artery was noted. Left internal jugular vein was significantly compressed by the lesion in the upper neck with complete nonvisualization of its upper part. Bony hyperostosis was noted on the left side of the skull base with erosion and enlargement of the left jugular foramen and hypoglossal canal (Figure 5).

**Figure 6:**
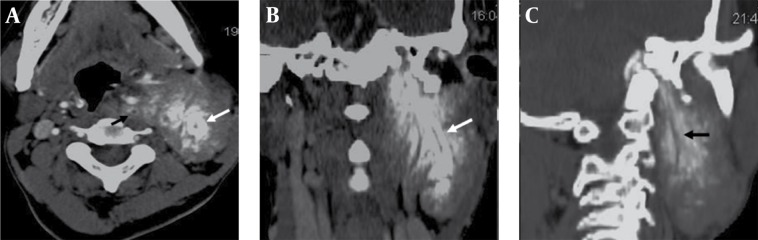
Axial (A), Coronal (B) and Oblique Sagittal (C) Reformatted Contrast Images Show Lineal Continuous Vertical Hypodense Strips Within the Calcified Area Giving the Tram Track Appearance (White And Black Arrows).

The atypical finding on CT scan was presence of two to three continuous linear vertical hypodensities with surrounding calcifications giving the ‘tram track appearance’ (Figure 6) within the lesion possibly suggesting encasement of the cranial nerves (correlated with history, clinical signs and skull base foramen involvement). In correlation with the anatomical course [2], it appears that the linear hypodensities with surrounding calcifications in relation to jugular foramen, extending along the posterolateral aspect of the left carotid artery indicates the left vagus nerve and the other with more dense surrounding calcification in relation to the hypoglossal canal extending inferiorly up to the level of oropharynx on the lateral aspect of the mass possibly indicates the left hypoglossal nerve. However, the glossopharyngeal nerve could not be appreciated clearly, which might be due to the lesser calcification around it.

**Figure 7:**
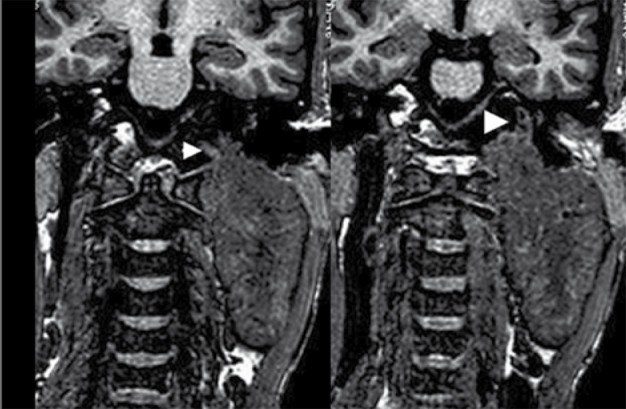
Coronal 3D T1 Weighted Reformatted Images Show Involvement of the Left Jugular and Hypoglossal Canal (Arrow Heads)

**Figure 8:**
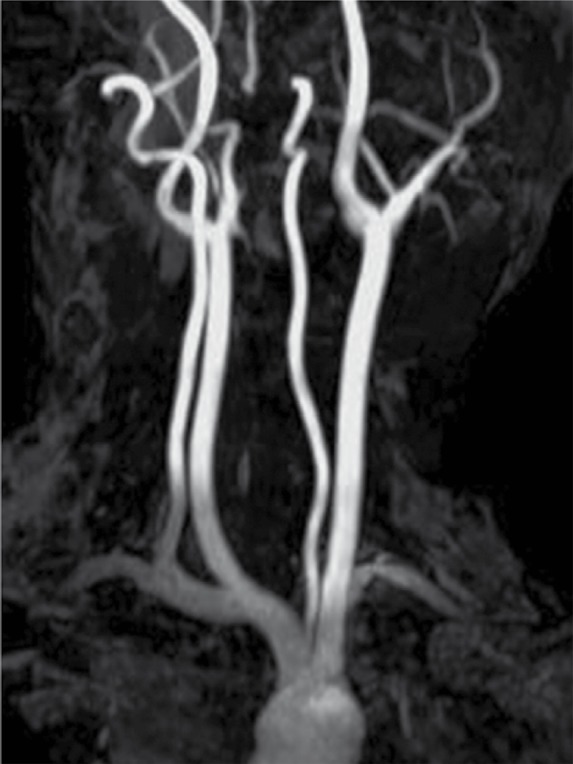
Coronal 3D TOF Image Shows Splaying of the Carotid Bifurcation.

**Figure 9:**
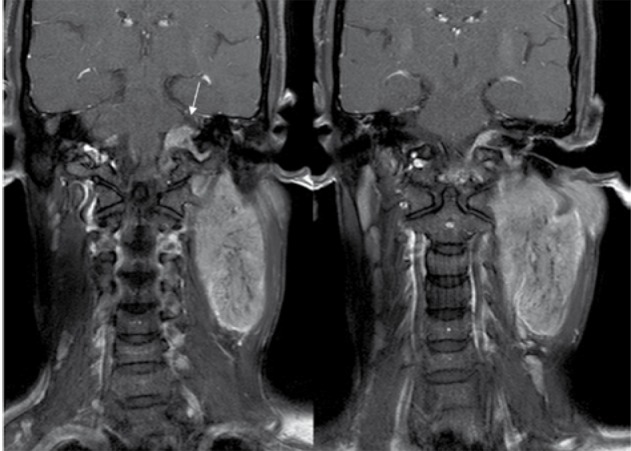
Post-Contrast Coronal 3D Gradient (TFE) T1 Weighted Images Show Involvement of the Left Hypoglossal Canal (Arrow) With Enhancing Intracranial Component.

**Figure 10:**
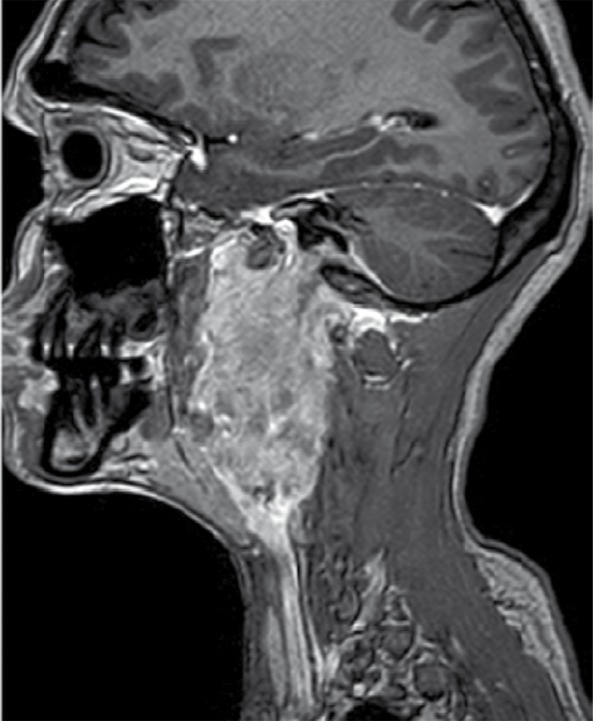
Post-Contrast Sagittal 3D Gradient (TFE) T1 Weighted Images Show Involvement of the Left Jugular Canal.

**Figure 11:**
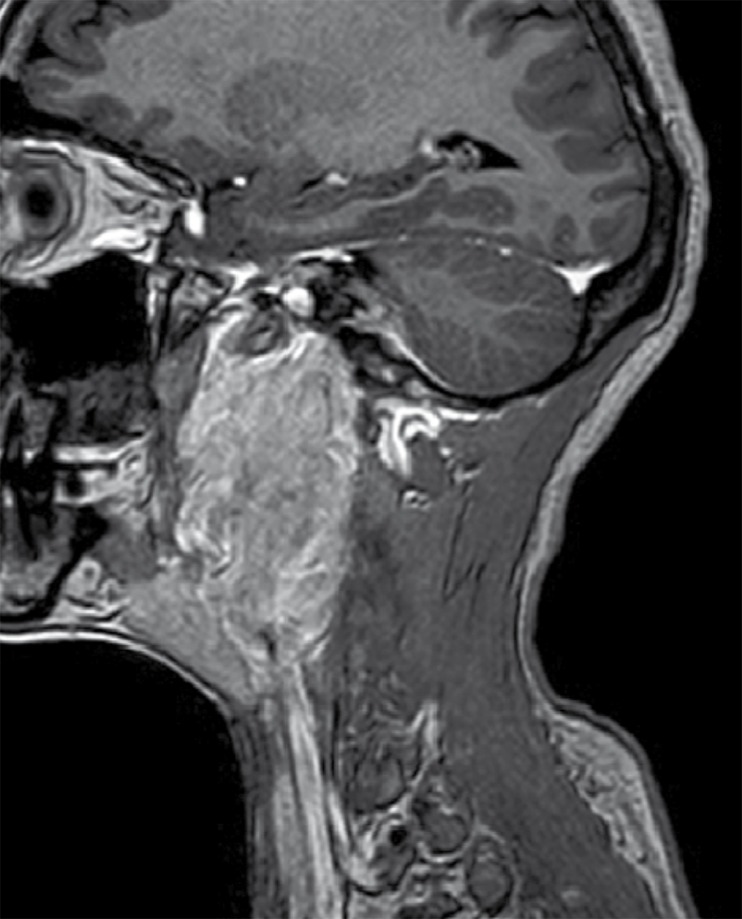
Post-Contrast Sagittal 3D Gradient (TFE) T1 Weighted Images Show Involvement of the Left Hypoglossal Canal With Enhancing Intracranial Component.

An MRI of the neck was performed to see the definitive status of vessels in relation to the lesion. Plain and contrast enhanced MRI with MRA (Figures 7, 8, 9, 10 & 11) revealed a heterogeneously enhancing altered signal intensity mass lesion in the left parapharyngeal region with a small enhancing intracranial extradural extension through the jugular foramen and hypoglossal canal. Gradient images showed multiple signal void foci within the lesion suggesting the presence of calcifications. Splaying of carotid vessels with encasement of the internal carotid artery up to the skull base and encasement with significant compression of the internal jugular vein was noted.

**Figure 12:**
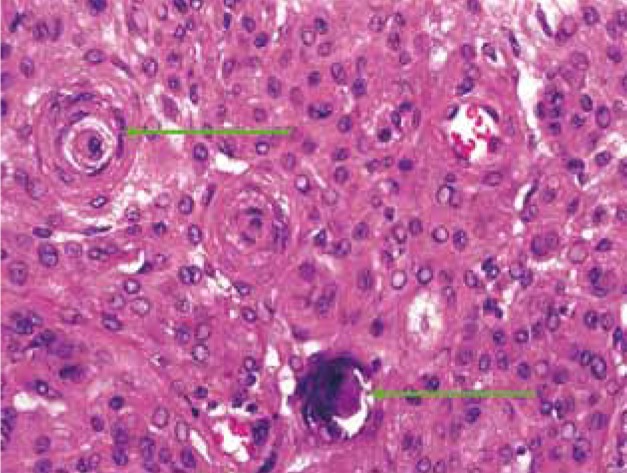
Histopathology Reveals Psammoma Bodies (Green Arrows) S/O Meningioma.

Biopsy of the mass lesion was carried out and histopathology revealed psammoma bodies suggestive of meningotheliomatous (transitional or psammomatous form) meningioma (Figure 12).

## 3. Discussion

Meningiomas are relatively common neoplasms of the central nervous system comprising about 18% of all primary intracranial tumors and about 25% of all primary intraspinal tumors. Meningiomas rarely extend out of their intracranial confines through the skull foramen to present as a neck mass. Extracranial extension of intracranial meningioma is rarely noted to occur into the confines of the orbit, scalp and paranasal sinuses. Extension and presentation into parapharyngeal space is exceedingly rare. Meningiomas outside the cranium constitute 2% of all meningiomas and primary extracranial meningiomas are even rarer [3][4]. Various other sites of extracranial meningiomas have also been quoted by Hollen and colleagues [5]. We report a rare case of extracranial, predominant parapharyngeal space meningioma presenting as a cervical mass with encasement of major carotid vessels and cranial nerves giving the ‘tram track appearance’.

Meningiomas arise from meningocytes of neuroectodermal origin, which accounts for the rarity of such tumors outside the cranial cavity and spinal cord. Arachnoid cell clusters may be seen at exits and in the sheaths of the cranial nerves [6]. Due to displacement of cells during the closure of fetal midline structures, heterotopic brain and meningeal tissue is known to occur in the midline of the head, neck and trunk. The meningiomas can arise from such ectopic locations [7]. However, meningiomas not related to midline structures and nerve sheaths are more difficult to explain.

Few authors [8] stated that subcutaneous meningiomas arise from the schwann cells because of the similarity between the cells of meningocytic meningioma and nevus cells which are derived from schwann cells [9].

Hoye and colleagues [10] divided the ectopic meningiomas into four categories as primary intracranial tumors with direct extracranial extension, tumors arising from arachnoid cell rests of cranial nerve sheaths with extracranial growth, tumors without any apparent connection with the foramina or cranial nerves and primary intracranial tumors with extracranial metastasis. According to Hoye’s classification, our case fits into the second category with meningioma in relation to the cranial nerves with extracranial parapharyngeal extension.

Though it is less common, the tram-track appearance may be seen in optic nerve sheath meningioma on unenhanced CT scan due to linear calcification of the sheath [11]. In the Sturge-Weber syndrome because of gyriform, curvilinear cortical calcifications that result from leptomeningeal vascular malformations, the tram-track sign may be seen on skull radiographs and CT [12]. In conjunction to these cases, we found similar findings in our case showing calcifications around the encased cranial nerves giving the tram track appearance on CT scan and MRI.

Four microscopic patterns of meningiomas are recognized [4]:

1) Syncytial type

2) Transitional or psammomatous form with a whorled pattern of spindle cells and psammoma bodies

3) Fibrous form

4) Angioblastic type

Most extracranial meningiomas are of syncytial or transitional form. Histopathology of our case revealed psammoma bodies suggestive of meningotheliomatous (transitional or psammomatous form) meningioma (Figure 12). Most of the meningiomas are treated surgically with the aim to remove the whole of the tumor along with the dural attachment and bone [13]. The most important prognostic factor in meningioma depends upon the completeness of the surgery. Significant post-operative morbidity and recurrence has been noted in few surgically treated extracranial meningiomas. However, surgical removal of parapharyngeal space meningioma is possible after occlusion of the ipsilateral internal carotid artery if the patient has good circulation from the contralateral carotid artery [14]. External beam radiation therapy may serve as palliative modality in inoperable or elderly patients or as an adjunct for control of residual disease after definitive surgery [15][16]. In the current case, angiography revealed insufficient blood supply from the contralateral side of the internal carotid artery and in view of significant postoperative morbidity, the patient was referred for radiotherapy. In conclusion, differentiation between various parapharyngeal masses at times may be difficult even with advanced imaging like CT and MRI. However, the demonstration of calcification, intracranial extension, contrast enhancement and dural component helps in suspecting the possibility of meningioma. The current case in correlation with history and clinical signs reveals that imaging may further help in defining the involvement of cranial nerves due to encasement (tram track appearance). Thus, one can predict the post-operative prognosis.
